# Metallo-Liposomes of Ruthenium Used as Promising Vectors of Genetic Material

**DOI:** 10.3390/pharmaceutics12050482

**Published:** 2020-05-25

**Authors:** José Antonio Lebrón, Francisco José Ostos, Manuel López-López, María Luisa Moyá, Carlos Sales, Encarnación García, Clara Beatriz García-Calderón, Margarita García-Calderón, María José Peña-Gómez, Iván V. Rosado, Fernando R. Balestra, Pablo Huertas, Pilar López-Cornejo

**Affiliations:** 1 Department of Physical Chemistry, Faculty of Chemistry, University of Seville, c/Prof. García González nº 1, 41012 Seville, Spain; jlebron@us.es (J.A.L.); fostos@us.es (F.J.O.); moya@us.es (M.L.M.); carsalsot@alum.us.es (C.S.); encarni_11_95@hotmail.com (E.G.); 2 Department of Chemical Engineering, Physical Chemistry and Materials Science, Faculty of Experimental Sciences, Campus de El Carmen, Avda. de las Fuerzas Armadas s/n, 21071 Huelva, Spain; manuel.lopez@diq.uhu.es; 3 Institute of Biomedicine of Seville (IBIS), University Hospital Virgen del Rocio/CSIC/University of Seville, Avda. Manuel Siurot s/n, 41013 Seville, Spain; claragarcia@us.es (C.B.G.-C.); marpengom3@alum.us.es (M.J.P.-G.); ivrosado@us.es (I.V.R.); 4 Department of Vegetal Biochemistry and Molecular Biology, Faculty of Chemistry, c/Prof. García González nº 1, 41012 Seville, Spain; marbioq@us.es; 5 Department of Genetics, University of Seville and Andalusian Center for Molecular Biology and Regenerative Medicine-CABIMER, 41092 Seville, Spain; fernando.balestra@cabimer.es (F.R.B.); pablo.huertas@cabimer.es (P.H.)

**Keywords:** non-toxic nanocarriers, specificity by cancer cells, gene therapy, transfection process, metallo-liposomes, ruthenium(II)-based lipids, metallo-liposomes

## Abstract

Gene therapy is a therapeutic process consisting of the transport of genetic material into cells. The design and preparation of novel carriers to transport DNA is an important research line in the medical field. Hybrid compounds such as metallo-liposomes, containing a mixture of lipids, were prepared and characterized. Cationic metal lipids derived from the [Ru(bpy)_3_]^2+^ complex, RuC11C11 or RuC19C19, both with different hydrophobic/lipophilic ratios, were mixed with the phospholipid DOPE. A relation between the size and the molar fraction α was found and a multidisciplinary study about the interaction between the metallo-liposomes and DNA was performed. The metallo-liposomes/DNA association was quantified and a relationship between K_app_ and α was obtained. Techniques such as AFM, SEM, zeta potential, dynamic light scattering and agarose gel electrophoresis demonstrated the formation of lipoplexes and showed the structure of the liposomes. L/D values corresponding to the polynucleotide’s condensation were estimated. In vitro assays proved the low cell toxicity of the metallo-liposomes, lower for normal cells than for cancer cell lines, and a good internalization into cells. The latter as well as the transfection measurements carried out with plasmid DNA pEGFP-C1 have demonstrated a good availability of the Ru(II)-based liposomes for being used as non-toxic nanovectors in gene therapy.

## 1. Introduction

Drugs have been always used to enhance life expectancy and to improve our health [1]. Generally, they are highly effective when they are used in vitro, but their indiscriminate use in vivo can provoke important damages in several internal organs and tissues of patients [2]. Nanocarriers with diverse structures and characteristics have been developed in the last decade to enhance the pharmacological action of drugs and to mitigate the side effects produced by the use of naked pharmaceuticals [3,4,5,6].

Nowadays, many studies about gene therapy are being carried out as a promising option to treat numerous acquired diseases such as cancer, AIDS or genetic disorders [7,8,9,10,11,12]. Biological defense mechanisms of the human body against the presence of foreign substances obligate the researchers to synthesize biocompatible and biodegradable nanosystems, also known as vectors, able to protect the genetic material. Such vectors can be viral or non-viral, the latter being usually less harmful to the health of the patients [13]. The more utilized non-viral vectors are nanoparticles, micelles, calixarenes, cyclodextrins and liposomes [14,15,16,17,18,19,20].

**Figure 1:**
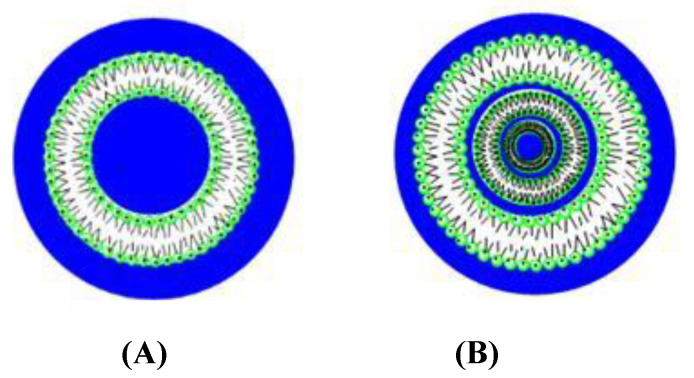
Structure of unilamellar (**A**) and multilamellar (**B**) liposomes.

Liposomes are spherical structures, similar to vesicles, which contain a nonpolar zone (a phospholipid bilayer) and an inner aqueous polar region (see Figure 1) [21]. Even though the formation of these systems is favored by the decrease of the contact among the phospholipid hydrophobic tails and the water molecules, it must be also noted that there are interactions that oppose such aggregation: (i) repulsive electrostatic interactions among the polar headgroups of the amphiphilic molecules (in the case of ionic liposomes), and (ii) steric hindrance effects due to the volume of such groups.

Liposomes are characterized according to the size and the number of bilayers forming the structure (Figure 1). The thickness of the bilayer depends on the length of the phospholipid non-polar tails. The charge of the nanostructures can be positive, negative or neutral [21,22,23].

According to the literature, cationic liposomes are good vectors to be used in gene therapy processes. They protect the genetic material from degradation by nucleases and overcome the electrostatic repulsion between the phosphate groups of the DNA backbone and negative groups of the lipid bilayer, favoring the delivery of genetic material into cells [24,25,26,27,28].

Given the importance of the use of metals in therapeutics and the diagnosis of medicinal chemistry [29], some vectors containing metals were prepared to transport genetic material. In this sense, Arroyo et al. [30] prepared liposomes containing copper (II) and zinc (II) complexes of 1-alkyl-1,4,7-triazacyclononane. Results showed a good DNA transfection efficiency in mammalian cells and a high ability of Cu-based liposomes to deliver DNA vaccine in murine experimental models. In general, it has been shown that electrochemical and photophysical properties of some metals provoke an improvement in the condensation of nucleic acids, favoring the endocytosis process and increasing the transfection efficiency [31,32,33,34].

**Figure 2:**
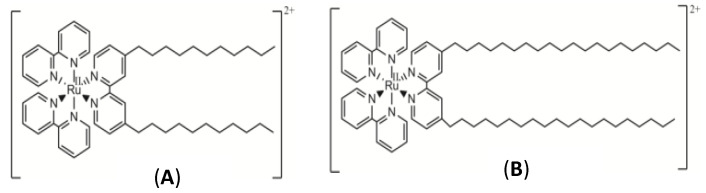
Structures of RuC11C11 (**A**) and RuC19C19 (**B**).

In this work, mixed unilamellar liposomes containing DOPE (1,2-dioleoyl-sn-glycero-3-phosphoethanolamine) and two cationic metallosurfactants derived from the 2,2′-trisbipyridine ruthenium (II) complex, with different length hydrocarbon tails (Figure 2), were prepared. The characterization of the Ru-based liposomes was carried out by using zeta potential and dynamic light scattering (DLS) measurements. Transmission electron microscopy (TEM) and scanning electron microscopy (SEM) images showed the structure of the vectors.

These liposomes were used as vectors of genetic material such as the plasmid DNA pEGFP-C1. A good internalization in diverse normal and cancer cell lines and a positive transfection process in the U2OS cell line was observed. A complete multidisciplinary study about the interactions liposome/DNA, that is, about the lipoplexes formation, was performed. Taking into account the fluorescence properties of the [Ru(bpy)_3_]^2+^ molecules anchored into the lipid membrane of the liposomes, fluorescence data were obtained in order to quantify the equilibrium binding constant of liposomes to DNA. To our knowledge, equilibrium association constants corresponding to the formation of lipoplexes have not been previously estimated.

Even though the encapsulation of DNA into liposomes, micelles, nanoparticles, and other non-viral vectors has been studied in numerous papers, the gene therapy phenomenon is still a priority goal in research, given the number of rare disorders in the population that need medical solutions. The presence of a fluorescent group into the vector structure facilities the diagnosis of the DNA internalization process without the addition of external dyes that can modify the characteristics of the lipid membrane.

## 2. Materials and Methods

### 2.1. Materials

Calf thymus DNA (ct-DNA) and Red-Safe were purchased from Sigma-Aldrich (Darmstadt, Germany) and iNtRON (Burlington, NJ, USA), respectively. The lipid 1,2-dioleoyl-sn-glycero-3-phosphoethanolamine (DOPE) was obtained from Avanti Polar Lipids (Alabaster, AL, USA). All reagents were of analytical grade (P.A.) and used without further purification. The concentration of the polynucleotide was given by phosphate groups and determined spectrophotometrically by using the known molar absorption data of 6600 mol^−1^ dm^3^ cm^−1^ at 260 nm [35]. The ratios of absorbance of the solutions at 260 and 280 nm (A_260_/A_280_) were in the range of 1.7–1.8, suggesting the absence of proteins [36]. Agarose gel electrophoresis using ethidium bromide gave an average number of base pairs per DNA molecule of 10,000 bp.

All solutions were prepared with distilled and deionized water (from a Millipore Milli-Q system, Darmstadt, Germany) with a conductivity value lower than 10^−6^ Sm^−1^. The pH of the solutions was maintained constant at a value of 7.4 with a HEPES buffer (I = 0.01 mol dm^−3^). All the measurements were done at 298.0 ± 0.1 K.

The syntheses of the ruthenium complexes [Ru(2,2′-bipy)_2_(4,4′-(C_11_H_23_)_2_-2,2′-bipy)]Cl_2_ (RuC11C11, see Figure 2A) and [Ru(2,2′-bipy)_2_(4,4′-(C_19_H_39_)_2_-2,2′-bipy)]Cl_2_ (RuC19C19, see Figure 2B) were previously described [37,38]. IR and NMR spectra and elemental analysis (C, H, N) were carried out for the characterization of these compounds. These measurements were recorded in the Research Services of the University of Seville (CITIUS) and data agreed with those previously reported [37,38].

### 2.2. Liposome Preparation

Liposomes were prepared by using the lipid thin-film hydration method [39,40]. First, appropriate quantities of DOPE and ruthenium-based lipids were dissolved in 2 mL chloroform. Different volumes of these solutions were mixed in order to obtain the lipid molar fraction (α) required. The organic solvent of the lipid mixture was evaporated by using a rotary evaporator for 50 min at 30 °C, obtaining a dry lipid film which was stored at 193 K for at least 24 h in order to avoid degradation of the phospholipid. After 24 h, the lipid film was hydrated with 2 mL of an aqueous solution of HEPES 10 mM (pH = 7.4) and subjected to 10 cycles of vortex (3 min/1200 rpm) and sonication (2 min, JP Selecta Ultrasons system 200 W and 50 kHz). Finally, the solution was vortexed for 2 h at room temperature.

The liposomes formed following this procedure are usually multilamellar and show a high polydispersity. In order to generate uniform populations of unilamellar liposomes with a homogenous size distribution (a low polydispersity), the extrusion of the liposomes solutions was carried out using a mini extruder from Avanti Polar Lipids and polycarbonate membranes of diameters of 100 nm and 200 nm from Whatman. The liposome sizes were found to be independent of the pore membrane diameter. This is due to the flexibility of the liposomes and to the equilibrium state towards which all liposomal solutions evolve. The liposome solutions were extruded 10 times. These solutions were maintained in the dark at 277 K for 24 h in order to get the complete stabilization of the nanosystems.

The composition of the cationic liposomes was expressed in molar fraction, *α*, defined as the molar fraction of cationic lipid (see Equation (1)).

(1) $$\alpha =\frac{n_{+}}{n_{o}+n_{+}}$$

*n_+_* and *n_o_* being the mole number of both the cationic (Ru) and zwitterionic (DOPE) lipids, respectively. All the concentrations are referred to as the total solution volume.

The composition (mole ratio) of the different liposomes prepared is listed in Table 1.

#### Lipoplex Formation

The composition of the lipoplexes prepared is given by the mass ratio L/D, defined as:

(2) $$\frac{L}{D}=\frac{\mathrm{total}\;\mathrm{lipid}\;\mathrm{mass}}{\mathrm{DNA}\;\mathrm{mass}}=\frac{\mathrm{DOPE}\;\mathrm{mass}\;+{\;T}^{+}\mathrm{mass}}{\;\mathrm{DNA}\;\mathrm{mass}}$$

T^+^ being the mass of the ruthenium-based lipids (RuC11C11 or RuC19C19). The mass of DNA was maintained constant in all the measurements at a value of 1.0 × 10^−4^ g, which corresponds to a concentration value of 8.1 × 10^−5^ mol dm^−3^ (given in base pair) in our working conditions. Appropriate volumes of both liposome and aqueous DNA solutions in HEPES 10 mM (pH = 7.4) were mixed to obtain the L/D values required at each α value studied.

### 2.3. Fluorescence Measurements

Emission spectra of the lipoplex solutions were performed to quantify the interaction between DNA and liposomes. Measurements were carried out in a Hitachi F-2500 spectrofluorimeter interfaced to a PC for the recording and handling of the spectra and connected to a flow Lauda thermostat to maintain the temperature at 298.0 ± 0.1 K. A standard fluorescence quartz cell of 10 mm path length was used. Emission intensities of the lipoplexes prepared (DOPE and Ru(II)-based liposomes + DNA) were measured at different L/D and α values. DNA concentration used was 8.1 × 10^−5^ mol dm^−3^. The excitation and emission wavelengths used were 456 nm and 600 nm, respectively.

### 2.4. Zeta-Potential Measurements

Zeta-potential (ζ) values were obtained measuring the electrophoretic mobility of the sample from the velocity of the particles using a laser Doppler velocimeter (LDV). The experiments were carried out with a Zetasizer Nano ZS Malvern Instrument Ltd. (Malvern, Worcestershire, UK) at 298.0 ± 0.1 K. A DTS1060 polycarbonate capillary cell was used. DNA concentration used was 8.1 × 10^−5^ mol dm^−3^. The measurements were repeated five times.

### 2.5. Dynamic Light-Scattering Measurements (DLS)

The size and the polydispersity index of the different liposomes prepared were obtained from DLS measurements by using a Zetasizer Nano ZS Malvern Instrument Ltd. (Worcestershire, UK). Samples were illuminated with a laser, at a fixed detection arrangement of 90° to the center of the cell area, and fluctuations in the intensities of the scattered light were analyzed. DNA concentration used was 8.1 × 10^−5^ mol dm^−3^. The obtained results were the average of 10 measurements. The temperature was maintained constant at 298.0 ± 0.1 K.

### 2.6. Circular Dichroism Spectra

Electronic circular dichroism (CD) spectra were recorded in a Biologic Mos-450 spectropolarimeter (Barcelona, Spain). A standard quartz cell of 10 mm path length was used. Spectra of DNA and lipoplexes were collected at different α and L/D values. DNA concentration used was 8.1 × 10^−5^ mol dm^−3^. Each spectrum was obtained from an average of 10 runs with a 5 min equilibration before each scan at 298.0 ± 0.1 K. The spectra obtained were expressed in terms of ellipticity, Ө_obs_.

### 2.7. Agarose Gel Electrophoresis

The gel was prepared in a buffer TAE (40 mM de Tris-acetate, 1 mM de EDTA) and 1% (*p/v*) agarose in a total volume of 180 mL. Red-Safe (20,000×, 10 μL) was used as a staining agent for the visualization of the double-stranded DNA. 20 μL of each sample (free DNA and lipoplexes at different α and L/D values, [DNA] = 8.1 × 10^−5^ mol dm^−3^) were mixed and homogenized with 5 μL of DNA loading buffer and loaded onto the precast agarose gel. Electrophoresis measurements were carried out at 90 V for a period of time of 90 min. Imaging treatment was performed by a transilluminator Ultima 16si Hoefer (Heidelberg, Germany).

### 2.8. Electronic Transsmision Microscopy (TEM)

TEM images of lipoplexes with different α and L/D values were recorded with a Zeiss Libra 120 scanning electron microscope at 80 kV. Samples were prepared by impregnation on a 300 mesh copper grid coated with collodion. Images were processed with a bottom-mounted TEM CCD camera and recorded with a resolution of 2048 × 2048 pixels. DNA concentration used was 2.1 × 10^−6^ mol dm^−3^.

### 2.9. Atomic Force Microscopy (AFM)

Atomic force microscopy is a technique used to obtain information about the structure of lipoplex complexes. AFM images were obtained with a Molecular Imaging PicoPlus 2500 AFM (Agilent Technologies, Las Rozas, Madrid). Silicon cantilevers (Model Pointprobe, Nanoworld) with a resonance frequency of around 240 KHz and nominal force constant 42 N/m were used. All images were recorded in the tapping mode, with scan speeds of about 0.5 Hz and data collection at 256 × 256 pixels.

DNA dilute solutions (1.5 μmol dm^−3^) were used due to the large size of the polynucleotide. Thirty microliter of lipoplex (or free DNA) solution was deposited onto modified mica, incubated for 30 min, washed with pure water and air-dried for AFM imaging. The modified mica surface was prepared by dropping a 0.1% (*v/v*) APTES aqueous solution onto a freshly cleaved mica surface. After 20 min, the surface was washed with ultra-pure water and air dried [41].

### 2.10. Scanning Electron Microscopy (SEM)

In order to confirm the structure of RuC11C11 and RuC19C19 liposomes, SEM images were recorded with a Zeiss Auriga Field Emission Scanning Electron Microscope (Carl Zeiss NTS GmbH, Oberkochen, Germany). Samples were prepared by encapsulating liposome solutions in agarose cylinders as follows: warm 6% agarose low melt point was loaded in the open tip of a typical insulin syringe and was allowed to gel at room temperature. A hole was made with the tip of a micropipette in the center of the capsule previously prepared and a few microliters of liposome solution were loaded into the hole. The tip of the open agarose capsule was then sealed with additional warm agarose to prevent leakage.

Encapsulated samples were fixed with 2.5% glutaraldehyde in 0.1 M sodium cacodylate buffer (pH 7.4) for 1 h at room temperature and post-fixed with 1% osmium tetraoxide in 0.1 M cacodylate buffer (pH 7.4) for 1 h at 4 °C. Samples were then dehydrated through a graded series of acetone solutions and dried using the critical point drying procedure in a Leica CDP300 critical point dryer. The agarose capsules containing the dried samples were sectioned in two parts mounted on aluminum stubs and sputter-coated with a 10 nm thick layer of gold/palladium. The open capsules were examined.

### 2.11. In Vitro Assays

In order to measure the cytotoxic activity of the liposomes of RuC11C11 and RuC19C19, in vitro measurements were carried out using the MTT assay [42]. Cell lines were plated out into 96 well plates at a density of 3000 cells per plate. Five human cancer cell lines and a normal cell line were used: A549 (adenocarcinomic human alveolar basal epithelial cell line), HepG2 (human liver cancer cell line), LS180 (adenocarcinomic human colonic epithelial cell line), MCF7 (breast cancer cell line) and RPE-1 (hTERT-immortalized retinal pigment epithelial cell line, normal cell line). Different doses of liposome solutions were added to the wells and the plate returned to the incubator for four more days. The medium was supplemented with different concentrations of liposomes of diverse α values. Later, they were pulsed with MTS (ROCHE). Cell viability was measured by luminometry according to the manufacturer’s instructions. Each dose point was measured in triplicate.

Taking into account the luminescence properties of the lipids containing the [Ru(bpy)_3_]^2+^ group, fluorescence microscopy was used to prove the intake of the liposomes prepared in diverse cancer and normal cell lines. In these assays, living cells were exposed to liposome solutions of RuC11C11 and RuC19C19 for 24 h. After the incubation time, cells were repeatedly washed with a PBS solution and mounted on coverslips using Prolong antifade mounting medium (Invitrogen, Ltd., Inchinnan, Renfrewshire Scotland). Images were taken with a DP72 camera attached to an Olympus BX61 fluorescence microscope using 40× magnification lenses, with an excitation filter at 470–490 nm and emission long-pass filter at 520 nm. Images were analyzed using cellSens Dimension software.

### 2.12. Transfection Assays

RuC11C11 and RuC19C19 liposomes were used as vehicles to transfect plasmid DNA into human tissue cultured cells. The plasmid pEGFP-C1 (from Clontech) was used for these experiments. This plasmid carries an enhanced GFP coding sequence with the necessary regulatory elements for constitutive expression of the gene in human cells. The U2OS cell line (human osteosarcoma cell, from ATCC^®^) was used because this is an easy-to-transfect cell line frequently used in human molecular and cellular biology studies [43]. In our experimental set up, 3 μg of plasmid DNA was added to a solution containing 180 μL of Opti-MEM (Gibco) and 60 μg of liposome buffered solution (HEPES 10 mM, pH = 7.4). The mixture was incubated at room temperature for 10 min and, subsequently, added it to a 50% confluent 6 cm plate with 3 mL of DMEM medium.

As a negative control, the cells were transfected with a mixture of plasmid DNA and Opti-MEM. As a positive control, we used FuGENE 6 transfection reagent according to the manufacturer’s protocol (i.e., 3 μg of plasmid DNA in 200 μL Opti-MEM plus 9 μL of FuGENE 6). Transfection efficiency was evaluated by flow cytometry with a FACSCalibur (BD). The excitation wavelength was 488 nm. A filter FL1 (515–545 nm) was used to detect the emission due to the transfected plasmid. Each point was measured in duplicate.

## 3. Results and Discussion

**Figure 3:**
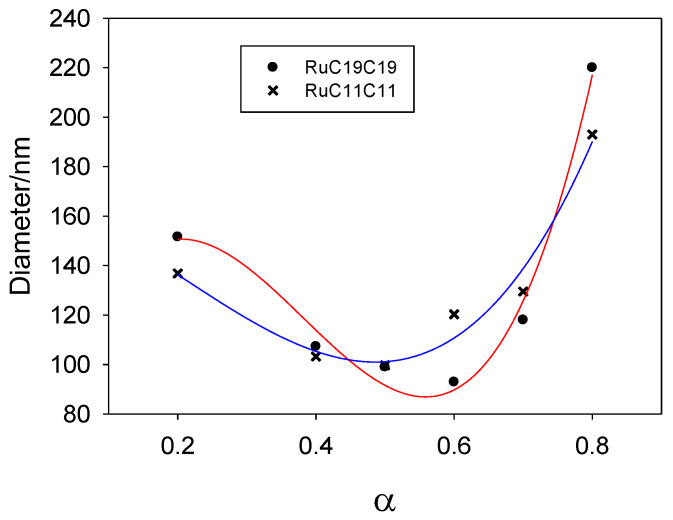
Size (diameter) of liposomes prepared from DOPE and Ru-based lipids (RuC11C11 or RuC19C19) at different compositions. Solid lines only indicate the trend obtained in the size with α value.

Mixed liposomes containing a lipid derived from the [Ru(bpy)_3_]^2+^ complex (RuC11C11 or RuC19C19, see Figure 2) and the phospholipid dioleoyl phosphatidylethanolamine, DOPE, were prepared at different α molar fractions. Appropriate quantities of lipids were dissolved in chloroform, the organic solvent was evaporated and, after 24 h at 173 K, the lipid bilayer was hydrated and extruded. The sizes of such liposomes were measured by dynamic light scattering. Figure 3 shows the liposome diameter values obtained for the molar fractions prepared. As can be seen, there is a dependence of the size on α for all the liposomes prepared, as well as a slight dependence on the Ru-based lipid used. The liposomes are formed by a mixture of lipids (Ru-lipid and DOPE). It must be noted that the interactions between the molecules of the diverse lipids are different; that is, the interaction between two Ru-lipid molecules is different than those between two DOPE molecules or between Ru-lipid and DOPE. This is due to the hydrophobic-lipophilic balance of each lipid molecule and provokes a variation in the size of the liposomes depending on their composition. According to Figure 3, there is an α value (~0.5 for RuC11C11 and ~0.6 for RuC19C19) at which the size of the liposomes is minimum. Taking into account that the two Ru-based lipids have the same cationic headgroups, these results demonstrate the influence of the lipophilic character of the lipids and/or of the hydrophobic interactions at work on the diameter of the nanostructure. According to the definition of the α parameter, an increase of this molar fraction means an increment of the amount of Ru-lipid in the liposomes. At low α values, the hydrophobic interactions among the hydrocarbon tails prevail over the electrostatic interactions taking place among the cationic headgroups of the lipids placed into the bilayer of the liposomes. The first decrease in the size observed when the quantity of Ru-lipid is augmented, keeping the quantity of DOPE constant, indicates that the hydrophobic interactions Ru-lipid/DOPE are stronger than those for Ru-lipid/Ru-lipid or DOPE/DOPE. A further increase in Ru-lipid in the bilayer of the liposomes, which is an increase in α molar fraction, causes an increment in the surface charge of the nanostructure due to the cationic headgroups of the metal complex. This results in an increase in the repulsive electrostatic interactions and, therefore, in the size of the liposomes as is seen in Figure 3. Therefore, the electrostatic interactions seem to mainly control the liposome size for a certain range of α values. The minimum size value would correspond to the α value where both the hydrophilic and the lipophilic contributions are compensated.

Figure 3 shows a weak liposome size dependence on the cationic lipid nature. This can also be explained following the same idea: a lower hydrophilic-lipophilic balance of the lipids leads to smaller liposomes. Taking into account that both Ru-based lipids have the same hydrophilic headgroups, the stronger lipophilic character of the RuC19C19 lipid will increase the hydrophobic interactions in the liposomes and, therefore, a higher molar concentration of Ru lipid will be needed to balance the hydrophilic-lipophilic ratio. This explains that the value of α corresponding to the smaller diameter of the liposomes would be higher for RuC19C19 than for RuC11C11, as is observed.

According to the idea of using Ru-based liposomes as nanovectors in gene therapy, it is necessary to study the interaction between these structures and DNA. A qualitative analysis of such binding was carried out from spectrophotometric measurements by taking advantage of the photophysical characteristics of the cationic lipid head groups. The fluorescence emission values corresponding to RuC11C11 and RuC19C19 liposomes in both the presence and the absence of nucleic acid was measured for different α and L/D (see Equation (2)) values. A decrease in the emission intensity was observed in all cases when the L/D ratio increases. Figure S1 shows the dependence of the fluorescence relative intensity with respect to the cationic lipid mass, EI/(RuC11C11- or RuC19C19-mass), versus the total lipid mass.

Taking into account the equilibrium process:

(3) $$\begin{array}{c} K\;\;\;\;\;\;\;\;\;\;\;\;\;\;\;\;\\ \mathrm{Liposome}\;+\;\mathrm{DNA}\;⇄\;\mathrm{Liposome}/\mathrm{DNA}\;(\mathrm{Lipoplex}) \end{array}$$

where K is the equilibrium constant corresponding to the binding of the liposome to DNA, the K value can be obtained by using the known Pseudophase model [44]. Bearing in mind that the ruthenium lipids (RuC11C11 and RuC19C19) in solution (free) and bound to the nucleic acid show different emission intensities, the following equation can be written:

(4) $$\mathrm{EI}\;=\;\frac{{\mathrm{EI}}_{\mathrm{free}}\;+\;{\mathrm{EI}}_{\mathrm{bound}}\;K\left[\mathrm{DNA}\right]}{1\;+\;K\left[\mathrm{DNA}\right]}$$

where EI is the total emission intensity of the RuC11C11 (or RuC19C19) liposomes in DNA solution, while EI_free_ and EI_bound_ represent the emission intensities of the Ru-based liposomes in solution and bound to DNA, respectively.

The EI values were measured at constant [DNA] and different total lipid concentrations. Bearing this in mind, the conventional Pseudophase model shown in Equation (4) must be modified.

According to the definition of the mass ratio L/D (see Equation (2)), one can obtain the nucleic acid mass as:

(5) $$\mathrm{DNA}\;\mathrm{mass}=\frac{\mathrm{total}\;\mathrm{lipid}\;\mathrm{mass}}{L/D}$$

If all the masses are referred to as the total volume, Equation (5) can be written as:

(6) $$\left[\mathrm{DNA}\right]=\frac{\left[\mathrm{total}\;\mathrm{lipid}\right]}{L/D}=\frac{\left[\mathrm{Liposome}\right]}{L/D}$$

By using Equations (4) and (6), the following Pseudophase model equation results:

(7) $$\mathrm{EI}\;=\;\frac{{\mathrm{EI}}_{\mathrm{free}}\;+\;{\mathrm{EI}}_{\mathrm{bound}}\;K_{\mathrm{app}}\;\left[\mathrm{Liposome}\right]}{1\;+\;K_{\mathrm{app}}\;\left[\mathrm{Liposome}\right]}$$

K_app_ being the apparent equilibrium binding constant defined as:

(8) $$K_{\mathrm{app}}=\frac{K}{L/D}$$

**Figure 4:**
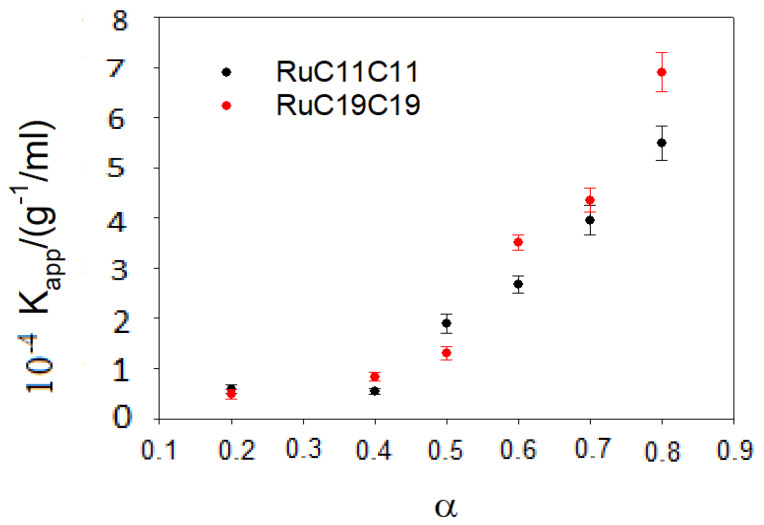
Dependence of the apparent equilibrium constant on the parameter α for the various prepared liposomes.

The K_app_ value can be obtained from a non-linear fit by using Equation (7). Figure 4 shows the results obtained for different α values. An increase in the parameter α, that is an increase in the cationic lipid (RuC11C11 or RuC19C19) concentration, provokes growth in the positive charge of the liposomes and, therefore, in the attractive electrostatic force with the negative phosphate groups of the DNA backbone. This will lead to an increase in the apparent equilibrium constant. On the other hand, the equilibrium constant does not depend practically on the length of the hydrocarbon tail. This fact indicates the importance of the electrostatic forces in the binding liposome/DNA.

The characterization of both the Ru-based liposomes and the lipoplexes was done from zeta potential (ζ) and light scattering measurements for different L/D and α values. The ζ values are collected in Figure S2. As can be seen, a sigmoidal behavior was obtained for both Ru lipids and for all α values studied. The zeta potential of the lipoplexes goes from negative to positive values until keeping constant for the highest α values. This behavior is usually related to a modification in the conformation of the nucleic acid. This will be discussed later in the text.

A displacement of the sigmoidal function for higher L/D values is also observed when α decreases. The crossing point of the sigmoidal function with the x-axis shown in Figure S2 (that is, the point corresponding to a ratio zeta potential values equal to zero) is reached at an L/D value at which the moles of both the positive and negative species in the lipoplexes are the same. This can be explained considering the definition of the parameter α (see Equation (1)), the positive charge of the liposome is higher, that is more positive, by increasing α and, therefore, a higher DNA concentration (or a lower L/D value) is needed to reach the electroneutrality in the lipoplex.

**Figure 5:**
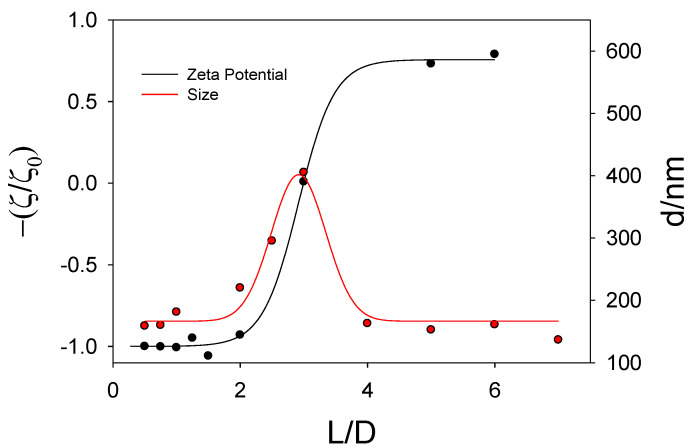
Plot of Z potential values (and size values) for lipoplexes containing RuC11C11 versus the parameter L/D at α = 0.5.

With respect to the dynamic light scattering measurements, the sizes of the lipoplexes show the tendency presented in Figure S3. The increase in size observed when the polynucleotide concentration is increased indicates the formation of lipoplexes. As was found in zeta potential measurements, such formation reduces the charge of the cationic liposomes due to the presence of the negative phosphate groups in the DNA backbone. This provokes neutralization of the aggregate charge and, therefore, they coalesce and precipitate in some cases. For each α value, the L/D value at which the maximum size is observed in Figure S3 is similar to the L/D value obtained when the neutralization of the system takes place (that is, the crossing point of the sigmoid function with the x-axis in Figure S2) as shown in Figure 5.

The decrease in size at high DNA concentrations (or low L/D values) is related to a conformational change in the DNA strands as will be discussed below.

**Figure 6:**
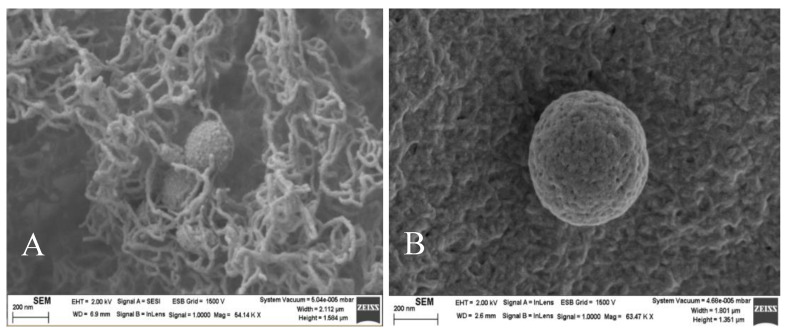
SEM images of RuC11C11- and RuC19C19-liposomes (**A**,**B**, respectively). α = 0.2. Scale bar = 200 nm.

The morphology of nanovectors is a significant factor to get good results in transfection efficiency [45]. Therefore, the structure of the liposomes was investigated from transmission electron microscopy. Liposomes and lipoplexes showed an approximately spherical structure (Figure S4) and the sizes estimated from these images were similar to those obtained from dynamic light scattering. The spherical structure of liposomes was confirmed from scanning electron microscopy (SEM) using a method of sample preparation with agarose cylinders (Figure 6).

Circular dichroism spectroscopy is a technique used to get information about conformational changes in polynucleotides. In order to go further in the study about the interactions liposome/DNA, circular dichroism spectra of DNA were run in the presence and absence of the RuC11C11 (or RuC19C19)-based liposomes. The B-form of DNA shows a characteristic spectrum in aqueous solution. This has a negative band, due to the ellipticity of the double chain centered at 242 nm, and a positive one centered at 280 nm due to base-stacking [46].

The CD spectra obtained (Figure S5) showed similar behavior for the two ruthenium-liposomes synthesized: a decrease of both negative and positive bands in the presence of DNA until their disappearance at the highest L/D values. These experimental observations indicate the formation of lipoplexes. Zeta potential, DLS and CD results make evident a change in the nucleic acid conformation, which is a requirement to cross the cell membrane, an essential process in gene therapy.

It is worth noting that the loss of band intensities in DNA CD spectra happens at lower L/D values for liposomes with RuC19C19 than with RuC11C11. As is observed in Figure 4 and Figure S3, liposomes containing RuC19C19 have a slightly higher size and a clearly lower superficial charge than those containing RuC11C11. According to the results obtained from circular dichroism spectroscopy, a lower charge/size ratio (as happened with RuC19C19 liposomes) favors the condensation process of the polynucleotide.

**Figure 7:**
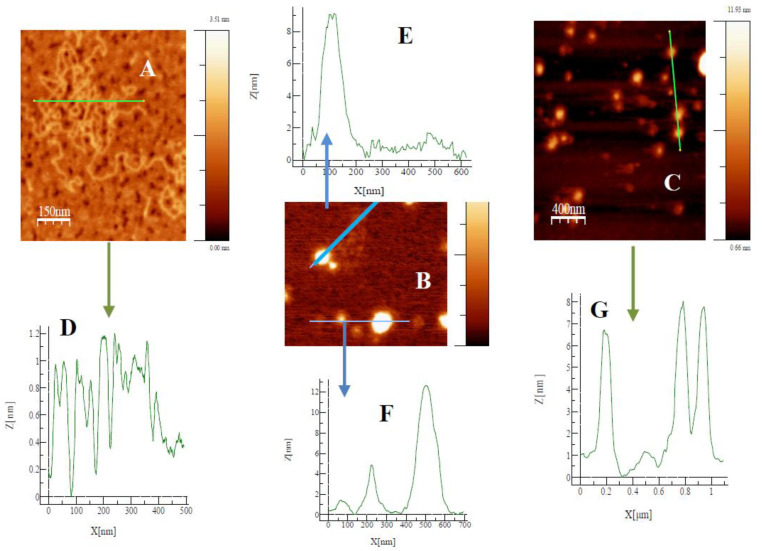
Topography AFM images for free DNA (**A**), and lipoplexes of RuC11C11 (**B**, α = 0.2 L/D = 6) and RuC19C19 (**C**, α = 0.2 L/D = 11). Profile of free DNA and lipoplexes (**D**–**G**).

The techniques of atomic force microscopy and gel electrophoresis confirmed the condensation process of the polynucleotide in the presence of the mixed metallo-liposomes of RuC11C11 (or RuC19C19) with DOPE. Results in AFM show the formation of lipoplexes (Figure 7) and the height profile of such images for the corresponding lines drawn. These profiles demonstrate the formation of such structures (the height value obtained is about 8–10 nm for lipoplexes and about 1 nm for free DNA). Electrophoresis proves that the charge inversion in DNA molecules takes place at lower L/D values for RuC19C19 liposomes than for RuC11C11 liposomes (Figure S6).

**Figure 8:**
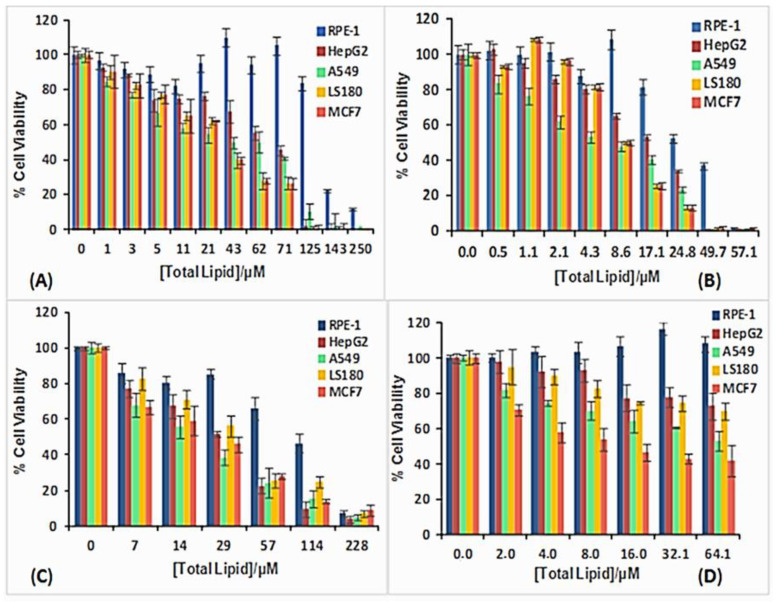
Cell viability of RuC11C11- and RuC19C19-based liposomes in different cell lines: (**A**) RuC11C11 α = 0.2, (**B**) RuC11C11 α = 0.8, (**C**) RuC19C19 α = 0.2, (**D**) RuC19C19 α = 0.8.

**Figure 9:**
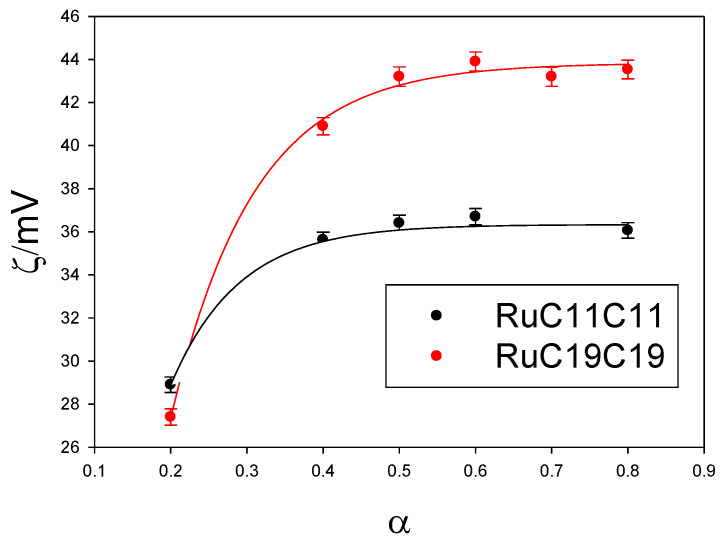
Dependence of zeta potential values of RuC11C11- or RuC19C19-based liposomes on α.

Given the importance of searching for new nanovectors, and the possibility of using metallo-liposomes with biomedical purposes, it is crucial to get information about the cytotoxicity and cell internalization properties of the Ru-based liposomes studied. Cytotoxicity data (Figure 8) showed low cell toxicity of both metal liposomes at low total lipid concentrations for all the cell lines studied. The cell viability decreases when α increases, due to an increment of the metal lipid concentration in the liposomes. Besides, metallo-liposomes with RuC11C11 seem to be more toxic than those with RuC19C19. This can be due to the charge of the liposomes that is lower for the nanostructures with RuC19C19 than for those with RuC11C11 (Figure 9). Therefore, the charge of the liposomes seems to exert more influence on the cytotoxicity than their size (larger for RuC19C19 liposomes).

It must also be noted that these metallo-liposomes are more cytotoxic for the cancer cells than for the normal cells. This specificity for cancer cells confirms the high capacity of these aggregates to be used in both gene therapy and cancer treatments.

**Figure 10:**
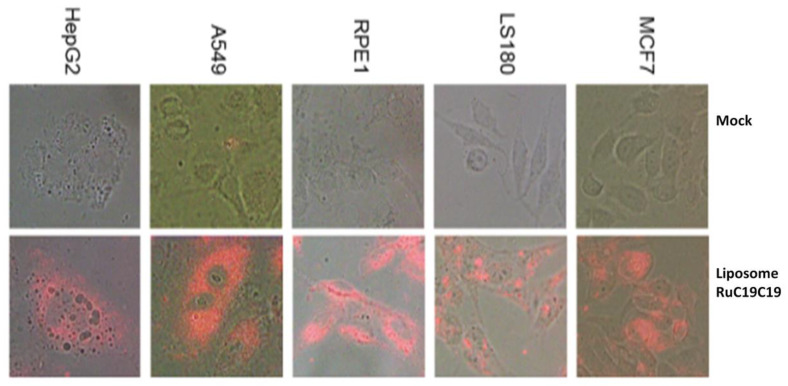
Fluorescence microscopy of the cell lines MCF7, LS180, HepG2, A549 and RPE-1 in the absence (mock) and presence of liposomes containing RuC19C19 and DOPE at α = 0.2 for 24 h, washed, fixed and mounted on coverslips. Magnification 40×.

For the effective action of these liposomes as nanocarriers of nucleic acids (or any other drug) they must be able to cross the cell membrane and penetrate into the cells. The process of cell membrane internalization was monitored for metallo-liposomes containing RuC11C11 or RuC19C19 in different cell lines (see Figure S7 and Figure 10, respectively). Results showed a good internalization of the nanovectors in all the normal and cancer cell lines studied. According to Gad et al. [47], polycations increase the membrane permeability. Therefore, the presence of ruthenium (II) in the nanostructure could increase the internalization process, as it happens with other metals such as Ca^2+^, Mg^2+^ and Zn^2+^ [31,32,33,34,47].

**Figure 11:**
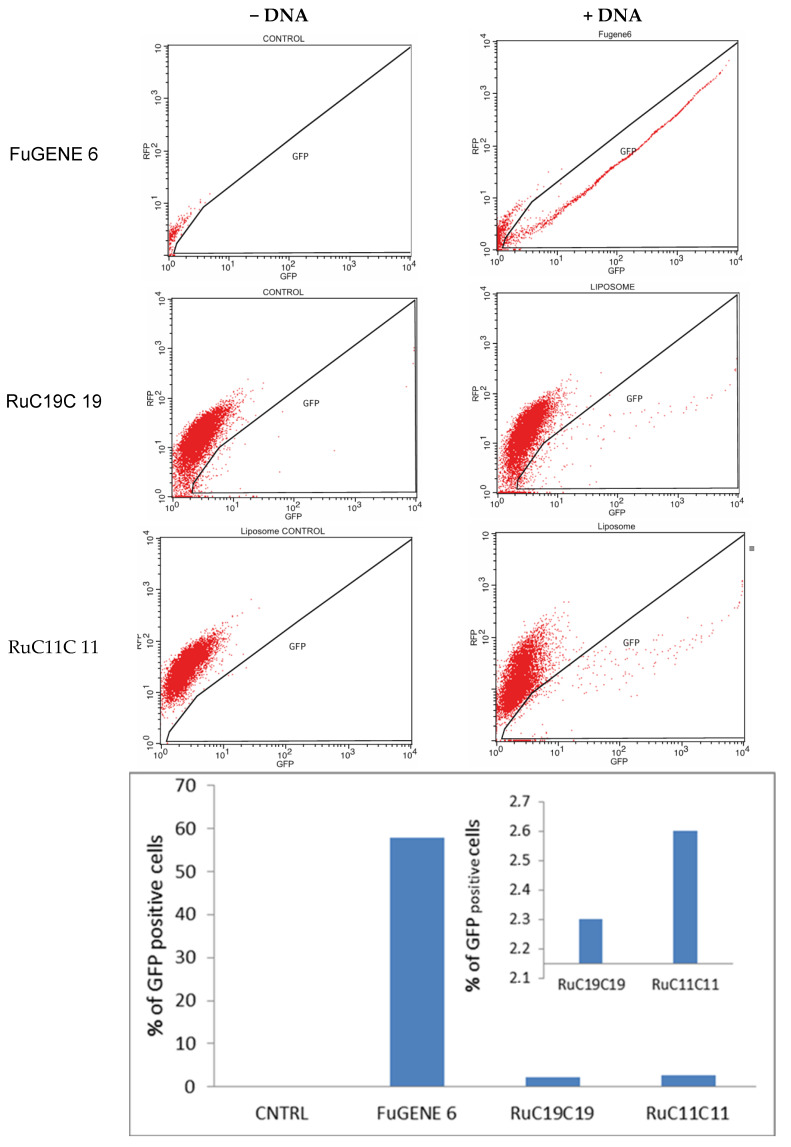
GFP expression of pEGFP-C1 in U2OS cells, using RuC11C11 and RuC19C19 liposomes at α = 0.2 and L/D = 20, sorted on the basis of fluorescence at 24 h, post-transfection. CNTRL represents the negative control. FuGENE 6 was used as a positive control.

The function of vectors in gene therapy is not only to internalize into cells, normally through an endocytosis process but also to favor the endosomal escape process. The transfection process of the plasmid pEGFP-C1 was performed on the human bone osteosarcoma epithelial cells (U2OS cells). Figure 11 shows the percent of GFP cells obtained for the liposome prepared. A transfection efficiency of 2–3% was obtained for both RuC11C11 and RuC19C19 lipoplexes at α = 0.2 and L/D = 20; lower than that obtained by using FuGENE 6 as a transfection reagent (approximately 60%).

The efficiency seems to be slightly higher for RuC11C11 than for RuC19C19. That is, an increase in the length of the Ru-lipid and, therefore a greater hydrophobic character of the lipid, decreases the transfection efficiency. Indeed, a greater hydrophobic character of the lipids present in the liposomes generates a more compact lipid bilayer. Therefore, the breaking of the liposome bilayer as well as the escape of the plasmid to the cytosol will be more difficult, as has been seen in this work. This result agrees with those obtained by Y. Xu, who says that shorter unsaturated hydrocarbon chains of lipids decrease the phase transition, increase the fluidity of the bilayer and favoring a higher intermembrane transfer rate and lipid mixing, resulting in potential disruption of the endosome and consequent DNA releasing from endosomal degradation [48]. Duangjit et al. [49] also found that surfactants with longer carbon chains may increase vesicle rigidity by inserting deeper into the bilayer.

Higher efficiency values were obtained by different researchers by using cationic lipids containing metals such as Ca^2+^, Mg^2+^ or Zn^2+^ [30,31,32,33,34,47]. The presence of polyamines or peptides containing several histidine residues, as well as the structure of the nanovector, were found to influence on the transfection efficiency.

In any case, the observation of a positive transfection efficiency for RuC11C11 and RuC19C19 liposomes (see Figure 11) compared to the negative control, confirms the possibility of using these liposomes as nanovectors in gene therapy. Further studies to optimize the transfection process and to elucidate the possible endocytosis pathways are in progress.

## 4. Conclusions

Cationic liposomes containing lipids derived from a ruthenium complex and the phospholipid DOPE were prepared. The metallo-liposomes were characterized and a relationship between the diameter and the parameter α was obtained. The hydrophilic-lipophilic balance of the lipids was found to influence the size of the liposomes. Lipoplexes were prepared and an apparent binding constant from the interaction metallo-liposome/DNA was obtained at a different molar fraction of the ruthenium lipids by using a modification of the Pseudophase Model. The characterization of both the liposomes and lipoplexes demonstrated the formation of spherical nanostructures in the presence and absence of DNA. Dichroism spectra of DNA, AFM images and gel electrophoresis assays showed the compaction of the polynucleotide, forming small nanocarriers of nucleic acids that can be used in gene therapy. The compaction happened at lower L/D values for RuC19C19-based lipoplexes than for RuC11C11-based lipoplexes. The size and superficial charge seem to be the cause of such behavior. Liposomes containing the ruthenium lipids RuC11C11 (or RuC19C19) seem to be less toxic for normal cells than for cancer cells and they show a good internalization in all the cell lines studied. Transfection measurements gave a low, but positive, transfection efficiency (2–3%); slightly higher for RuC11C11 than for RuC19C19 liposomes. This was explained considering the length of the unsaturated hydrocarbon tails of the lipids that form liposomes: the fluidity of the lipid bilayer is larger for shorter lipids than for longer lipids. Results in this work demonstrate that liposomes derived from both ruthenium and DOPE lipids are promising new vectors for DNA delivery. Future work will be performed in order to optimize and improve their transfection efficiency. Given the potential importance of the subject, and the good results found, our research group continues working in this research line.

## Figures and Tables

**Table 1:** Liposome composition (mole ratio). Ru-lipid: RuC11C11 or RuC19C19.

A	0.2	0.4	0.5	0.6	0.7	0.8
**Ru-lipid:DOPE** **(mol:mol)**	1:4	1:1.5	1:1	1:0.67	1:0.43	1:0.25

## Supplementary Materials

The following figures are available online at https://www.mdpi.com/1999-4923/12/5/482/s1. Figure S1: Plot of the relative fluorescence intensity, with respect to the cationic lipid mass, versus the total lipid mass at different α values. RuC11C11: (A) α = 0.2 and (B) α = 0.8. RuC19C19: (C) α = 0.2 and (D) α = 0.8. Lines show the best fit obtained by using equation 7; Figure S2: Plot of the relative zeta potential (ζ and ζ0 being the zeta potential values in the presence and absence of liposome) versus the L/D ratio for different α values: () α = 0.2, () α = 0.4, () α = 0.5, () α = 0.6, () α = 0.7 y () α = 0.8; Figure S3: Plot of the lipoplex diameter (nm) versus the L/D ratio for different α values: () α = 0.2, () α = 0.4, () α = 0.5, () α = 0.6, () α = 0.7 y () α = 0.8; Figure S4: TEM images of RuC11C11- and RuC19C19-liposomes (A and B, respectively) and RuC11C11- and RuC19C19-lipoplexes (C and D, respectively). A and B: α = 0.2, C and D: α = 0.2 L/D = 11. [DNA] = 2.1 × 10^−6^ mol dm^−3^; Figure S5: CD spectra of DNA ([DNA] = 8.1 × 10^−5^ mol dm^−3^) in the presence and absence of RuC11C11-liposomes (A) and RuC19C19-liposomes (B) at different α values. Figure S6: Agarose gel electrophoresis of free DNA, free liposomes (L) and lipoplexes at different α and L/D values. RuC11C11 lipoplexes at α = 0.2 (A) and α = 0.8 (B); and RuC19C19 lipoplexes at α = 0.2 (C) and α = 0.8 (D); Figure S7: Fluorescence microscopy of the cell lines MCF7, LS180, HepG2, A549 and RPE-1 exposed to liposomes containing RuC11C11 at α = 0.2 for 24 h, washed, fixed and mounted on coverslips.
