# Supplementary material for: Metallo-Liposomes of Ruthenium Used as Promising Vectors of Genetic Material
Source: Pharmaceutics. 2020 May 25;12(5):482. doi: 10.3390/pharmaceutics12050482 (PMC7284339; doi:10.3390/pharmaceutics12050482)
Supplement: Supplementary file 1 [file pharmaceutics-12-00482-s001.pdf]

pharmaceutics

MDPI

Supplementary Materials: Metallo-Liposomes of Ruthenium Used as Promising Vectors of Genetic Material

José Antonio Lebrón, Francisco José Ostos, Manuel López-López, María Luisa Moyá, Carlos Sales, Encarnación García, Clara Beatriz García-Calderón, Margarita García-Calderón, María José Peña-Gómez, Iván V. Rosado, Fernando R. Balestra, Pablo Huertas and Pilar López-Cornejo *

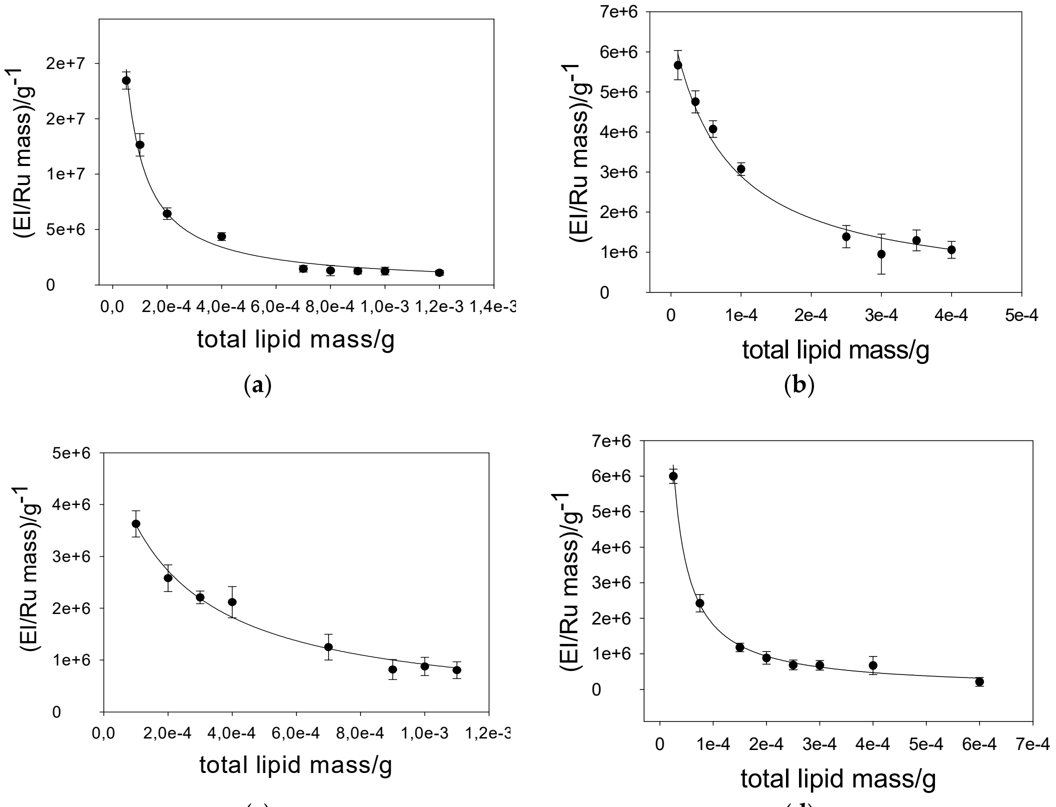

Figure S1. Plot of the relative fluorescence intensity versus the total lipid mass at different α values. RuC11C11: A) α=0.2 and B) α=0.8. RuC19C19: C) α=0.2 and D) α=0.8. Lines show the best fit obtained by using equation 7.

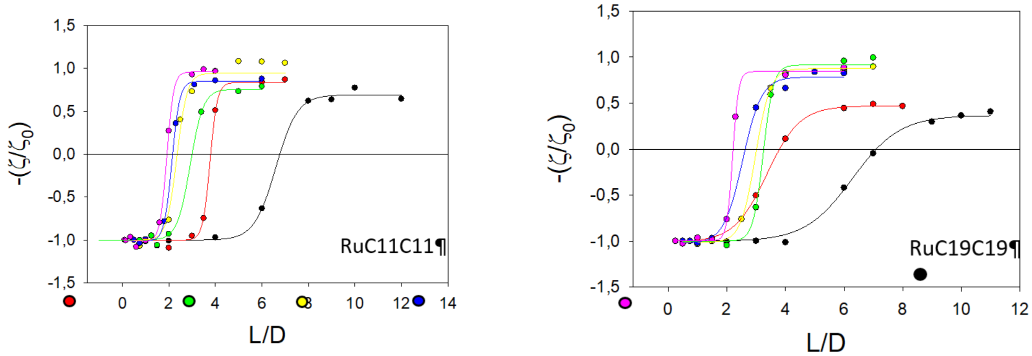

Figure S2. Plot of the relative zeta potential (ζ and ζ_0 being the zeta potential values in the presence and absence of liposome) versus the L/D ratio for different α values: (●) α =0.2, (●) α = 0.4, (●) α= 0.5, (●) α = 0.6, (●) α =0.7 y (●) α =0.8.

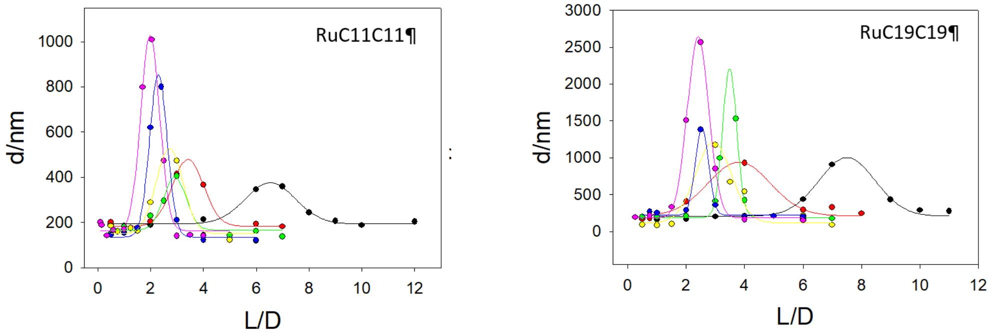

Figure S3. Plot of the lipoplex diameter (nm) versus the L/D ratio for different α values: (●) α =0.2, (●) α = 0.4, (●) α= 0.5, (●) α = 0.6, (●) α =0.7 y (●) α =0.8.

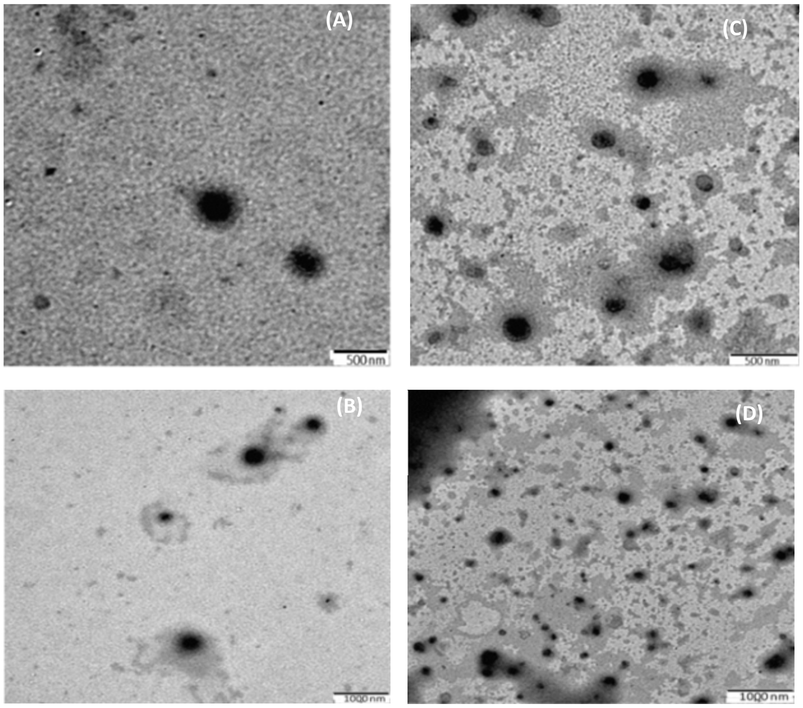

Figure S4. TEM images of RuC11C11- and RuC19C19-liposomes (**A** and **B**, respectively) and RuC11C11- and RuC19C19-lipoplexes (**C** and **D**, respectively). A and B: α = 0.2, C and D: α = 0.2 L/D=11. [DNA]=2.1 × 10^{-6} mol dm^{-3}.

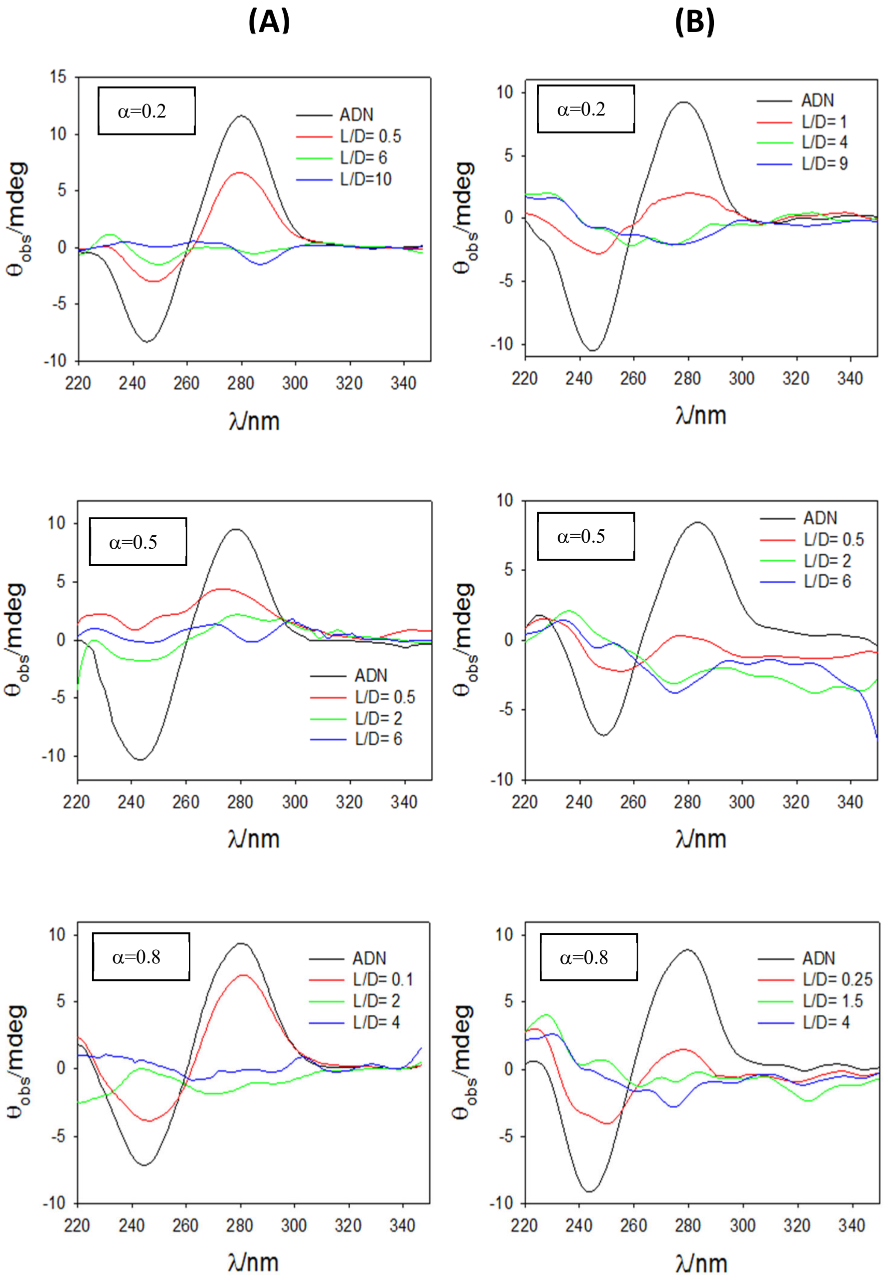

Figure S5. CD spectra of DNA ([DNA] = 8.1 × 10^{-5} mol dm^{-3}) in the presence and absence of RuC11C11-liposomes (**A**) and RuC19C19-liposomes (**B**) at different α values.

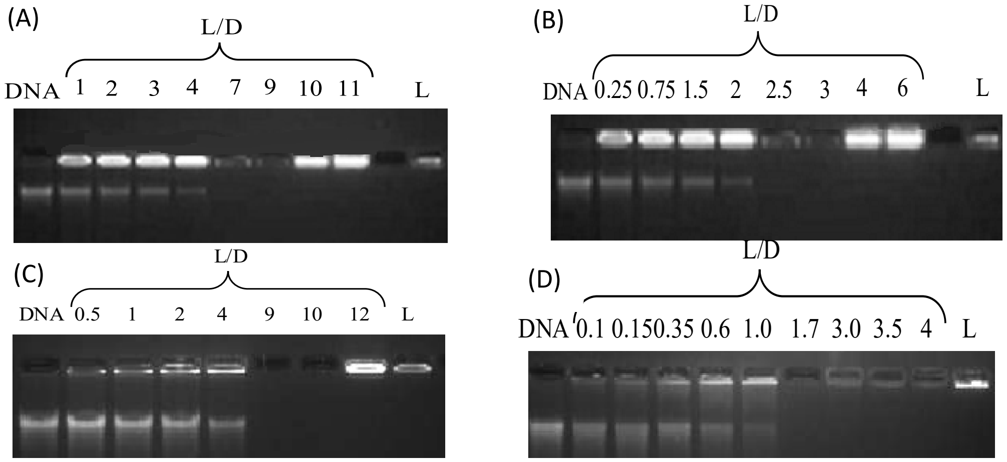

Figure S6. Agarose gel electrophoresis of free DNA, free liposomes (L) and lipoplexes at different α and L/D values. RuC11C11 lipoplexes at α = 0.2 (**A**) and α = 0.8 (**B**); and RuC19C19 lipoplexes at α = 0.2 (**C**) and α = 0.8 (**D**).

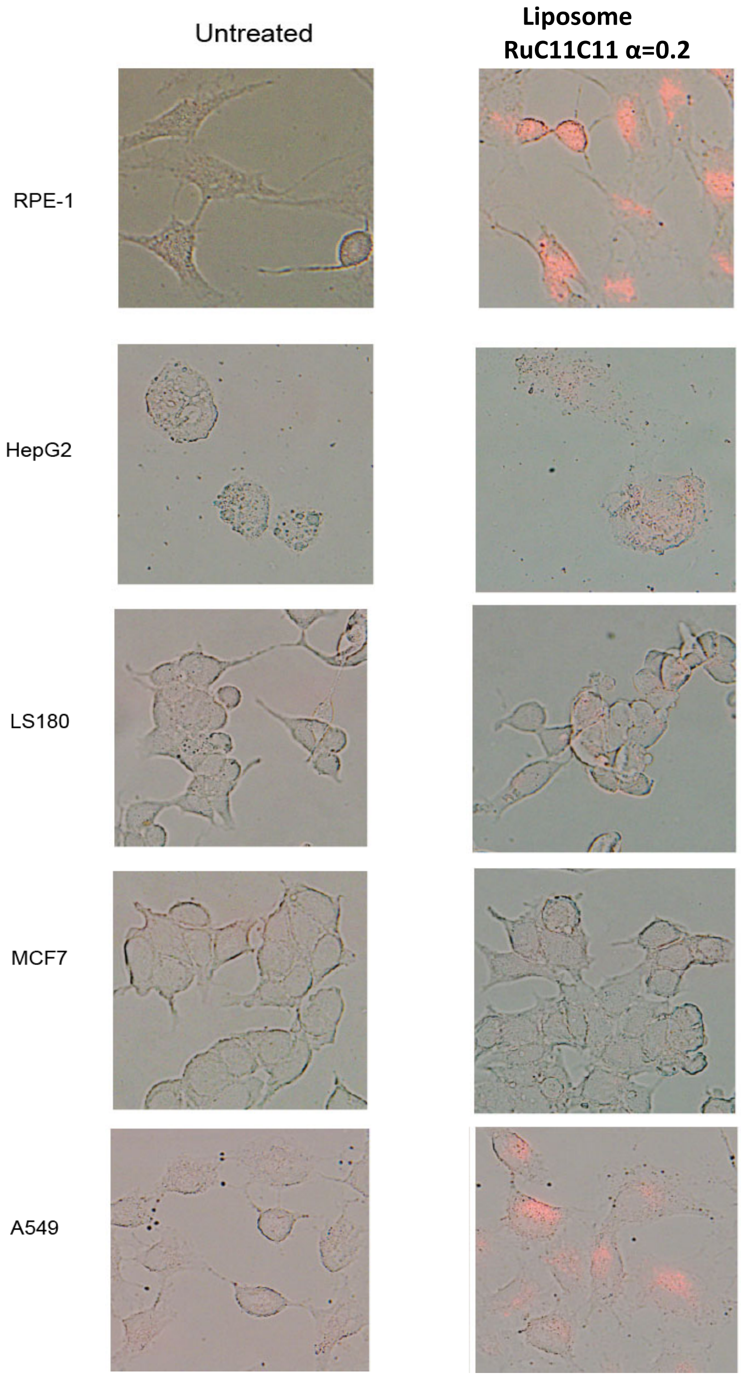

Figure S7. Fluorescence microscopy of the cell lines MCF7, LS180, HepG2, A549 and RPE-1 in the absence (mock) and presence of liposomes containing RuC11C11 at α= 0.2 for 24 hours, washed, fixed and mounted on coverslips. Magnification 40×.
